# Inhibitory Control in Age-Related Hearing Loss

**DOI:** 10.1093/geroni/igab046.1849

**Published:** 2021-12-17

**Authors:** Shraddha Shende, Lydia Nguyen, Grace Rochford, Raksha Mudar

**Affiliations:** 1 University of Illinois Urbana-Champaign, Champaign, Illinois, United States; 2 iN, 2.L, Denver, Colorado, United States; 3 University of Illinois-Urbana Champaign, Champaign, Illinois, United States

## Abstract

Inhibitory control involves suppression of goal irrelevant information and responses. Emerging evidence suggests alterations in inhibitory control in individuals with age-related hearing loss (ARHL), however, few have specifically studied individuals with mild ARHL. We examined behavioral and event related potential (ERP) differences between 14 older adults with mild ARHL (mean age: 69.43 ± 7.73 years) and 14 age- and education-matched normal hearing (NH, mean age: 66.57 ± 5.70 years) controls on two Go/NoGo tasks: a simpler, basic categorization task (Single Car; SC) and a more difficult, superordinate categorization task (Object Animal; OA). The SC task consisted of exemplars of a single car and dog, and the OA task consisted of exemplars of multiple objects and animals. Participants were required to respond to Go trials (e.g., cars in SC) with a button press, and withhold responses on NoGo trials (e.g., dogs in SC task). Behavioral results revealed that ARHL group had worse accuracy on NoGo trials on the OA task, but not on the SC task. ARHL group had longer N2 latency for NoGo compared to Go trials in the simpler SC Task, but no differences were observed on the OA task between Go and NoGo trials. These findings suggest that more prolonged neural effort in the ARHL group on the SC task NoGo trials may have contributed to their ability to successfully suppress false alarms comparable to the NH group. Overall, these findings provide evidence for behavioral and neural changes in inhibitory control in ARHL.

